# Can postoperative pain be predicted? New parameter: analgesia nociception index

**DOI:** 10.3906/sag-1811-194

**Published:** 2020-02-13

**Authors:** Ali Şefik KÖPRÜLÜ, Ali HASPOLAT, Yaşar Gökhan GÜL, Nurşen TANRIKULU

**Affiliations:** 1 Department of Anesthesiology and Intensive Care, Faculty of Medicine, İstanbul Yeni Yüzyıl University, İstanbul Turkey; 2 Anesthesia and Intensive Care Clinics, İstanbul Şişli Vocational High School, İstanbul Turkey; 3 Anesthesiology Clinics, Kolan Bayrampaşa Hospital, İstanbul Turkey

**Keywords:** General anesthesia, postoperative pain, intra/postoperative monitoring, analgesia nociception index

## Abstract

**Background/aim:**

The Analgesia Nociception Index (ANI) is a new method of identifying nociception-analgesia balance. In this study, we investigate the correlation between the ANI and numeric rating scale (NRS) values immediately before and after extubation. The NRS values were recorded in the postanesthesia care unit, in a group of patients who underwent laparoscopic cholecystectomy, with the aim of evaluating the potential use of ANI values in the prediction of postoperative pain levels.

**Materials and methods:**

The ANI and NRS values, heartbeat rate (HR), systolic and diastolic arterial pressure (SAP/DAP), and oxygen saturation (SpO2) values of the patients were recorded into three groups based on the initial NRS values recorded in the postanesthesia care unit (group I: NRS ≤ 3, group II: NRS 4–6, group III: NRS ≥ 7). Patients whose ANI values were lower than 47, considered as the pain threshold, and the groups to which these patients belonged were also recorded.

**Results:**

Statistically significant increases were noted in HR, SAP, and DAP after extubation, while there was no significant change in ANI values. A weak correlation was identified between the ANI and NRS values of all patient groups.

**Conclusion:**

We failed to identify a correlation between ANI and NRS values before and after extubation. Previous studies suggested that the ANI provides more valuable information in anesthetized patients, whereas our findings show that it is ineffective in the prediction of potential postoperative pain.

## 1. Introduction

A pain-free life is one of the fundamental human rights, and controlling pain, particularly in the postoperative period, is of vital importance for patient comfort and the following recovery period. Approximately 20%–40% of patients suffer from severe postoperative pain that begins immediately after surgery [1]. Such severe pain occurs not only after long-lasting and complicated surgical operations, but may also be seen after various minor or moderate surgeries, such as tonsillectomy, hemorrhoidectomy, laparoscopic cholecystectomy, and appendectomy [1]. 

The optimum method for assessing pain severity in postanesthesia care units (PACUs), where patients are monitored immediately after an operation, is still a matter of debate. In conscious and cooperative patients who have come out of anesthesia, commonly used assessment tools include visual analog scale (VAS/0–100), verbal rating scales (VRS/1–5), and numerical rating scales (NRS/0–10) [2,3,4]. While VAS ≤ 30 and NRS ≤ 3 are considered as evidence of analgesia or tolerable pain, scores of VAS ≥ 70 and NRS ≥ 7 are considered to indicate severe pain [4]. There are, however, large groups of patients who are unable to communicate (pediatrics, geriatrics, patients with communication disorders, unconscious patients, etc.), who face a risk of receiving insufficient pain treatment despite all measures. Methods such as skin conductivity and pupillary reflex measurements have been tested in these patients to detect levels of pain [5–7]. 

In recent years, the Analgesia Nociception Index (ANI monitor, MetroDoloris Medical Systems, Lille, France), which assesses the nociception-analgesia balance by measuring the parasympathetic system tonus, has emerged as a new method for the numerical and objective assessment of the sufficiency of perioperative analgesia [8,9]. The ANI measures the duration between two R waves within heart rate variations by filtering based on the variations in respiratory cycles, and it provides a numeric measure of parasympathetic tonus that varies between (p∑) 0 and 100. Based on this index, values of 50 and above indicate sufficient anesthesia, 30–50 indicate moderate pain, and values lower than 30 indicate severe pain [9–12]. Over the last few years, researchers have reported preliminary findings suggesting that the severity of potential postoperative pain can be predicted objectively, irrespective of the physician’s subjective assessment, based on ANI values recorded immediately after surgery [13], and these data may even allow the prediction of the severity of early postoperative pain [14,15]. 

In the present study, we investigate whether or not a correlation exists between the ANI values recorded at the completion of an operation and immediately before and after extubation and the NRS values recorded in the PACU in a group of patients who underwent laparoscopic cholecystectomy, with the goal of evaluating the potential use of ANI values for the prediction of postoperative pain levels. 

## 2. Materials and methods

We gained approval for the study from the ethics committee (İstanbul Arel University/69396709-050.01.01) to study with patients who provided informed consent for the use of all their medical data in medical research, as long as their identity was kept confidential. Thirty-six patients who underwent laparoscopic cholecystectomies under sevoflurane/remifentanil anesthesia at our hospital between 1 May and 15 August 2018, who were monitored using the ANI and who were assessed for postoperative pain based on the NRS in the PACU, were included in the study. 

The study exclusion criteria included patients with ASA score other than ASA I–II, age below 18 years or above 75 years, apparent cardiac disorders (primary arrhythmia, ECG abnormalities, coronary ischemia, heart failure, etc.) and marked preoperative pain.

Additionally, we excluded patients with an autonomous nervous system abnormality (epilepsy, previous CVE, etc.), chronic hypertensive patients taking beta-blockers, patients with a diagnosis of diabetes mellitus, and patients who had been given ketamine, atropine, beta-blockers, or other vasoactive substances at any time during surgery. 

In order to minimize potential differences in pain levels based on the surgical technique used, all surgical operations were performed by the same surgical team when feasible. The routine general anesthesia protocol of our clinics for intraabdominal laparoscopic surgeries was followed for all patients who agreed to take part in the study. The monitoring procedures included a 5-electrode 2-channel ECG, oxygen saturation (SpO2), noninvasive arterial blood pressure, end-tidal carbon dioxide (ETCO2), body temperature monitoring, and the recording of the ANI using an ANI monitor (ANI/MetroDoloris Medical Systems, Lille, France). As the only difference from the routine anesthesia protocol, spontaneous resolution of neuromuscular blocker activity was awaited or a specific antidote (sugammadex) was administered to the patients in the study group during the postoperative period. In order to conclude that neuromuscular activity was resolved spontaneously, we expected to observe that the patient had sufficient respiration, coughing, and swallowing of secretions. Additionally, the patient had to have eyes open, keeping the head lifted for more than 5 s, keeping the mouth firmly shut, and positively reacting to the tongue test. Sugammadex was used based on the decision of an anesthesiologist with at least 10 years of experience to eliminate risks of residual muscle weakness, which is a major risk for postoperative respiratory complications. Patients who were given an acetylcholinesterase inhibitor and/or anticholinergic due to clinical need were excluded from the study. As is the case for all patients scheduled for surgery in our clinics, the patients in study group were informed of the postoperative use of the NRS and were told how the postoperative pain evaluation would be performed during preoperative visits, as per the routine clinical protocol. Premedication was performed with midazolam (0.03 mg/kg) and fentanyl (1 mgr/kg) in the operating room. Propofol (1.5 mg/kg) was used for anesthesia induction, while orotracheal intubation and muscle relaxation were facilitated by rocuronium bromide (0.6 mg/kg). Sevoflurane (0.8–1.2 MAC) and an air/O2 mixture to maintain fiO2 of 50% and remifentanil infusion (0.04 μg/kg/min) for analgesia were performed for anesthesia maintenance. Volume-control mode with 6–8 mL/kg tidal volume to maintain SpO2 between 96% and 100% and EtCO2 between 35% and 40% was preferred for ventilation. If necessary, additional muscle relaxation by rocuronium bromide (0.15 mg/kg) was done. Our choice for perioperative fluid infusion was crystalloids (4 mL/kg/h, unless there was an additional indication). Pneumoperitoneum was maintained by maximum carbon-dioxide gas insufflation pressure of ≤15 mmHg. The body temperature was kept stable between 36.5 °C and 37.0 °C by intravenous fluids at body temperature and external heating during the perioperative period. Sugammadex (2–4 mg/kg) was used if clinically indicated for those patients who did not meet the above-mentioned criteria.

The ANI values of the patients were recorded immediately prior to extubation in the operating room and after extubation in the PACU, and pain scores were recorded based on the NRS within 10 min of the admission of the patients to the PACU, as well as hemodynamic data [heartbeat rate (HR), systolic arterial pressure (SAP), diastolic arterial pressure (DAP), and SpO2 levels] of the patients at the same time points. As per the clinical indication, patients with an NRS of >3 were given 1 mg/kg tramadol (30 min of slow IV infusion in 100 mL of 5% dextrose) for postoperative analgesia. Patients who experienced no problems during routine monitoring were transferred to the surgery inpatient wards.

For data evaluation, the patients were classified into 3 groups [group I: NRS ≤ 3 (17 patients), group II: NRS 4–6 (11 patients), group III: NRS ≥ 7 (8 patients)] according to the initial NRS values recorded in the PACU. The demographic parameters, durations of operations, and ANI values were compared between the three groups. The Patients whose ANI values were lower than 47, considered as the pain threshold, and the groups to which these patients belonged were recorded [13,14]. Correlations between the ANI values recorded before extubation and at the time of coming out of anesthesia, immediately after the completion of surgery, and after extubation in the PACU, and the NRS values recorded in the PACU, were analyzed. 

### 2.1. Statistical analysis

Qualitative data were compared using the chi-square test, while quantitative data were compared between the groups using Student’s t-test for normally distributed parameters and the Mann–Whitney U-test for nonnormally distributed parameters. For within-group comparisons, a paired t-test was used for normally distributed parameters, and the Wilcoxon signed ranks test was used for nonnormally distributed parameters. For all tests, P < 0.05 was considered statistically significant, while P < 0.001 was considered highly significant. Correlations between the ANI values and NRS were investigated by Pearson’s correlation test, and r values were used to evaluate statistical significance. Based on these analyses, r values of 0.00–0.29 indicated weak, 0.30–0.49 indicated low, 0.50–0.69 indicated moderate, 0.70–0.89 indicated strong, and 0.90–1.00 indicated very strong correlations. 

## 3. Results

A total of 36 ASA I–II patients, including 21 women and 15 men, were included in the study. The mean age of the study population was 45.33 ± 12.43 years, and the mean body weight, height, and BMI of patients was 74.22 ± 12.64 kg, 167.55 ± 7.72 cm, and 26.21 ± 4.49 kg/m2, respectively. The average duration of operation was 68.41 ± 15.81 min, and following the operation, the NRS values of the patients recorded in the PACU were ≤3 in 17 (47.22%) (group I) and between 4 and 6 in 11 patients (30.56%) (group II). The NRS was ≥7 in eight patients (22.22%) (group III). Table 1 summarizes the demographic data, the duration of operation, and NRS values in all groups. 

**Table 1:** The demographic findings of all patients and duration of operation.

Study group	n = 36
ASA I/II (n)	17/19
Sex (F/M) (n)	21/15
Age (years)	45.33 ± 12.43
Weight (kg)	74.22± 12.64
Height (cm)	167.55 ± 7.72
BMI	26.21 ± 4.49
Duration of operation (min) (top)	68.41 ± 15.81
NRS ≤3 (group I)	17 (47.22%)
NRS 4–6 (group II)	11 (30.56%)
NRS ≥7 (group III)	8 (22.23%)

ANI: Analgesia Nociception Index; ASA: American Society of Anesthesiologists; PS: physical status; BMI: body mass index; NRS: numerical rating scale.

During the stable phases of perioperative anesthesia, i.e. during the intraoperative period in which only routine surgical and anesthetic procedures were applied without any unexpected events, the mean HR, SAP, DAP, and ANI values of the patients were 65.31 ± 16.76 beats/min, 95.67 ± 15.06 mmHg, 59.72 ± 12.34 mmHg, and 70.78 ± 16.66, respectively. Before extubation, the mean HR, SAP, and DAP increased to 70.44 ± 15.42 beats/min, 111.97 ± 18.89 mmHg, and 71.44 ± 14.39 mmHg, respectively, whereas the mean ANI decreased to 59.81 ± 14.46. While there was a partial increase in hemodynamic parameters and a partial decrease in ANI, these differences were not statistically significant (P ≥ 0.05). Following extubation, the mean HR, SAP, and DAP increased but ANI decreased with values of 79.03 ± 14.37 beats/min, 127.14 ± 18.48 mmHg, 75.47 ± 12.16 mmHg, and 60.16 ± 12.61, respectively. The increases in all three hemodynamic parameters were statistically significant (P < 0.05). While there was a decrease in the ANI, the difference was not statistically significant (P > 0.05). Table 2 summarizes these findings.

**Table 2:** The hemodynamic parameters and ANI values in measurement periods.

		Heartbeat rate (beats/min)	Systolic pressure (mmHg)	Diastolic pressure (mmHg)	ANI
Perioperative		65.31 ± 16.76	95.67 ± 15.06	59.72 ± 12.34	70.78 ± 16.66
Extubation	Before	70.44 ± 15.42	111.97 ± 18.89	71.44 ± 14.39	59.81 ± 14.46	After	79.03 ± 14.371	127.14 ± 18.482	75.47 ± 12.163	60.72 ± 14.63

HRperiop/HRaft.ext P1 = 0.006; SAPperiop/SAPaft.ext P2 = 0.0415; DAPperi.op/DAPaft.ext P3= 0.009. P < 0.05, statistically significant difference.

Group I comprised a total of 17 patients (47.22%), including five ASA I and 12 ASA II patients whose postoperative NRS was ≤3. The group was made up of 7 women and 10 men, with a mean age of 45.41 ± 11.53 years, a mean body weight of 74.41 ± 12.12 kg, a mean height of 168.24 ± 7.44 cm, and a mean BMI of 26.04 ± 4.09 kg/m2. The mean duration of operation in this patient group was 67.26 ± 11.56 min, and the mean ANI values recorded before and after extubation were 58.47 ± 15.32 and 62.71 ± 15.61, respectively. Group II consisted of 11 patients (30.56%), including 7 women and 4 men, whose NRS was between 4 and 6. The mean age of these patients was 42.36 ± 8.74 years, and 9 patients were ASA I while the remaining 2 patients were ASA II. The mean body weight, height, and BMI of patients in this group were 73.57 ± 11.09 kg, 168.27 ± 7.01 cm, and 25.71 ± 2.91 kg/m2, respectively. The mean duration of operation was 69.48 ± 14.37 min, and the mean ANI values recorded before and after extubation were 62.73 ± 15.49 and 61.73 ± 14.32, respectively. A total of 8 patients (22.23%), including three ASA I and five ASA II, with 7 women and 1 man, made up group III with NRS of ≥7. The mean age of the patients in this group was 47.87 ± 14.94 years and mean body weight, height, and BMI were 78.21 ± 11.82 kg, 165.13 ± 9.41 cm, and 27.34 ± 3.39 kg/m2, respectively. The mean duration of operation was 68.45 ± 13.76 min, and the mean ANI values recorded before and after extubation were 58.50 ± 15.16 and 55.13 ± 13.54, respectively. While these values were numerically lower than in the other groups, the differences were not statistically significant (P ≥ 0.05). The other demographic findings, duration of operation, and ANI values were also not significantly different between the three groups. In addition, the ANI measurements were lower than the pain threshold level (47 for ANI) in 6 patients before extubation (2 patients in group I, 1 patient in group II, and 3 patients in group III), and also they were lower in 7 patients after extubation (4 patients in group I, 1 patient in group II, and 2 patients in group III). Only one patient who had an ANI value lower than 47 before extubation had a similar ANI value after extubation. All the relevant values are presented in Table 3. When postextubation NRS-ANI correlations were investigated between the three groups, classified based on NRS values, the correlations with preextubation values were found to be “weak” for group I (r = 0.016), “weak” for group II (r = –0.286), and also “weak” for group III (r = –0.293), representing the NRS ≥ 7 group. When the postextubation ANI values were considered, the correlation coefficients indicated weak correlations for group I (r = 0.135), group II (r = –0.069), and group III (r = –0.290). The ANI and NRS values of the patients and the correlations between these values are presented in Table 4 for preextubation and in Table 5 for postextubation time points, and the Figure summarizes the NRS-ANI distribution and correlation curves. 

**Table 3:** The comparison of the demographics and other characteristics between patients classified based on pain severity.

Study group “pain” level	Group I (NRS ≤ 3)	Group II (NRS 4–6)	Group III(NRS ≥ 7)
Number of patients and percentage, n (%)	17 (%)	11 (%)	8 (%)
ASA I/II, n	5/12	9/2	3/5
Sex (F/M), n	7/10	7/4	7/1
Age, years	45.41 ± 11.53	42.36 ± 8.74	47.87 ± 14.94
Weight, kg	74.41 ± 12.12	73.57 ± 11.09	78.21 ± 11.82
Height (cm), mean ± SD	168.24 ± 7.44	168.27 ± 7.01	165.13 ± 9.41
Body mass index (BMI), mean ± SD	26.04 ± 4.09	25.71 ± 2.91	27.34 ± 3.39
Duration of operation (min), mean ± SD	67.26 ± 11.56	69.48 ± 14.37	68.45 ± 13.76
ANI (before extubation), mean ± SD	58.47 ± 15.32	62.73 ± 15.49	58.50 ± 15.16
ANI before extubation, <47 patients	2	1	3
ANI after extubation, mean ± SD	62.71 ± 15.61	61.73 ± 14.32	55.13 ± 13.54
ANI before extubation, <47 patients	4	1	2

SD: Standard deviation; ANI: Analgesia Nociception Index; ASA: American Society of Anesthesiologists; PS: physical status; BMI: body mass index; NRS: numerical rating scale.

**Table 4:** The preextubation ANI values of patients classified based on pain level and NRS/ANI correlation

	Group I - NRS ≤ 3		Group II - NRS 4–6		Group III - NRS ≥ 7	
	ANI	NRS	ANI	NRS	ANI	NRS
1	50	1	63	6	52	10
2	71	2	78	5	76	8
3	33	1	76	5	42	9
4	55	2	54	6	70	7
5	61	2	43	6	49	9
6	72	2	48	6	75	8
7	59	1	67	4	52	7
8	86	2	57	5	81	7
9	39	2	48	6		
10	72	3	77	5		
11	38	3	94	4		
12	55	3				
13	57	3				
14	54	3				
15	89	2				
16	59	3				
17	44	3				
Mean ± SD	58.47 ± 15.33		62.73 ± 15.49		58.5 ± 15.16	
r	0.016 (weak)		–0.286 (weak)		–0.293 (weak)	

r = (0.00–0.29: weak, 0.30–0.49: low, 0.50–0.69: moderate, 0.70–0.89: strong, 0.90–1.00: very strong) relation.

**Table 5:** The postextubation ANI values of patients classified based on pain level and NRS/ANI correlation.

	Group I - NRS ≤ 3		Group II - NRS 4–6		Group III - NRS ≥ 7	
	ANI	NRS	ANI	NRS	ANI	NRS
1	42	1	51	6	53	10
2	58	2	82	5	44	8
3	48	1	83	5	61	9
4	97	2	53	6	55	7
5	79	2	51	6	44	9
6	60	2	57	6	50	8
7	67	1	56	4	85	7
8	91	2	51	5	63	7
9	52	2	65	6		
10	70	3	86	5		
11	61	3	44	4		
12	50	3				
13	52	3				
14	71	3				
15	38	2				
16	59	3				
17	71	3				
Mean ± SD	62.71 ± 15.61		61.73 ± 14.32		55.13 ± 13.54	
r	0.135 (weak)		–0.069 (weak)		–0.290 (weak)	

r = (0.00–0.29: weak, 0.30–0.49: low, 0.50–0.69: moderate, 0.70–0.89: strong, 0.90–1.00: very strong) relation.

**Figure 1:**
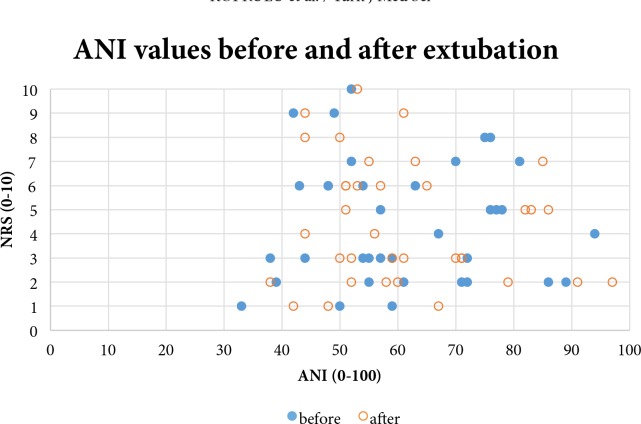
The distribution of ANI and NRS values before and after extubation.

## 4. Discussion

The ANI is commonly thought of as a numerical and objective indicator of perioperative analgesia levels [15], although some authors have suggested that potential hemodynamic changes can be predicted based on perioperatively monitored ANI values [16], while there have been further studies reporting the contrary [12,18,19]. Overall, almost all of those studies indicated that the ANI values may be in line with clinical status, even during spontaneous respiration in sedated patients under the influence of anesthesia [17–19]. In addition, some studies tested the use of the ANI to detect the level of analgesia in pediatric patients, and the results of such preliminary studies were found to be positive [20,21].

Extending these general considerations, some authors argued that the severity of potential postoperative pain can be measured objectively based on the ANI values recorded at the end of the perioperative period [13], and even that the level of pain can be predicted based on these values, therefore allowing the early identification of severe pain risk and the making of effective interventions accordingly [14,15]. 

In the present study, while 35.48% of the patients had mild pain that required no analgesia, 64.52% required additional postoperative analgesics. Of all the patients, 22.23% had NRS ≥ 7, or in other words, severe pain. This considerably high rate detected during a relatively minimally invasive operation such as laparoscopic surgery is proof of how important the concerns and actions to find a solution are. The rate identified in this study is consistent with previously reported rates in the literature [1]. 

In the present study, we failed to identify a correlation between the ANI and postoperative NRS, as suggested previously by some authors, and no significant correlation was noted between the ANI values recorded before and after extubation and the NRS values in any of our patient groups. In all patient groups, only a weak correlation was identified between the ANI and NRS values. In addition, despite some limitations, our findings suggested that the ANI was ineffective in the prediction of potential postoperative pain, and these findings are consistent with those of some other researchers [22,23].

The patient exclusion criteria used in this study are similar to those used previously in almost all studies on this issue, although there are some differences between these studies in terms of the types of operation, the anesthesia protocols, and the study design [23,24]. While the ANI values recorded immediately before extubation were considered as the measurement parameter in one of the first studies reporting a positive outcome [14], another study took into account the ANI values recorded after extubation [13]. In the present study, we investigated the ANI values recorded at both time points, and in this respect, we believe that our study is more inclusive. 

In the present study, the ANI values recorded in the postoperative period immediately after extubation were found to be slightly lower than those recorded during the perioperative period. However, the difference between these values was not statistically significant. This can be easily attributed to the status of patients, who are still partially under the influence of anesthetic medications, and who are gradually coming out of anesthesia and the effects of analgesic medications. There have also been several studies reporting that the ANI values were markedly higher during deep sedation when compared to the awake periods, although this relationship is not proportional to the degree of sedation [22]. This is most probably also true for our patient group. 

The ANI values also did not change significantly after extubation. In addition, the variations in ANI values were not parallel to the NRC values at either measurement time point. Low or high ANI values could be recorded in patients with apparent pain or in patients without any pain at all. In addition, aside from one patient, none of those who had an ANI value below 47 before extubation and were theoretically assumed to have pain had an ANI value below the threshold of 47 after extubation. 

Other than pain, it is also possible that other factors that might affect the sympathetic nervous system, such as nausea, vomiting, agitation, anxiety, voice, and others, may be involved and negatively affect the parameters measured in PACUs. All of these factors may affect HR and may consequently influence the ANI scores. Even authors who suggested that the ANI can be used for the prediction of postoperative pain underlined these potential effects [14]. In addition, there are authors who may be considered pioneers in supporting the use of the ANI during the perioperative period also, who reported that the effectiveness of the ANI is markedly decreased in conscious patients [17]. More recently, the results reported in two studies including healthy conscious subjects identified no direct relationship between NRS and ANI, highlighting the potential differences in individual responses [25,26]. In another study performed in 2016, Jess et al. reported that the ANI could not differentiate between painful, painless, and fake stimuli in conscious subjects and failed to detect nociception in conscious patients, while the values were affected by stress and emotional status. Based on all these findings, Jess et al. argued that the ANI lacked the ability to assess pain severity in conscious and stressed individuals [25]. In 2017, Issa et al. revealed a very weak negative correlation between the ANI and NRS in healthy conscious individuals, and they did not recommend the use of the ANI in an emergency unit or intensive care setting [26]. Indeed, there have been some researchers who contributed to the development of the ANI, such as De Jonckheere, who recently highlighted the relationship between the ANI and emotional status and even recommended use of the ANI for the detection of parasympathetic changes in different emotional moods [27,28]. Based on our findings, we also believe that the ANI is better able to reflect pain under anesthetic conditions, whereas the values recorded after the patients come out of anesthesia are rather complicated due to the interactions between other confounding factors and do not correctly reflect the balance between the sympathetic and parasympathetic nervous systems. On the other hand, we are currently engaged in further studies on the ANI in the different patient groups in our clinics.

Apart from all the above-described factors, ANI scores may also be affected by the applied anesthetic medication and the duration of exposure to such medications. While the mean duration of operation was approximately 65 min in all patient groups, the durations of operations in previous positive or negative studies varied between 30 and 180 min. Although there were studies that did not provide any clear data on this parameter, none of these studies considered the duration of operation to be a parameter with a direct effect on the outcomes [29]. We believe that as duration of operation affects the total time of exposure to anesthetic agents and, therefore, the total dose of analgesic medications, it should be considered as making a considerable difference. 

One of the reasons for the conflicting results reported by previous studies may be the differences in study designs. In the majority of studies reporting positive outcomes, total intravenous anesthesia (TIVA - propofol) was the preferred method of anesthesia, and these studies demonstrated that the performance of the ANI was better when using TIVA (propofol) than when using halogen-based agents [13,14,22,29]. Propofol and halogen-based agents have different effects on HR [30] and heart rate control via the baroreflex pathway [31]. While propofol decreases parasympathetic tonus in parallel to the degree of hypnosis, halogen-based volatile anesthetics have no such effects [32]. On the other hand, desflurane and isoflurane have been shown to decrease neural system activity in total, and to have a direct effect on sympathetic/parasympathetic balance [33]. Moreover, there have been other studies demonstrating that, compared to TIVA, high sympathetic activity that may affect HR and elevated plasma noradrenaline levels can be seen after sevoflurane-based anesthesia [34]. Still, it is apparent that halogen-based agents (sevoflurane, desflurane) are used much more often in daily anesthesia practice when compared to TIVA. In the present study, sevoflurane was used for the maintenance of anesthesia in all patients, and for this reason, we believe that our anesthesia protocol more realistically reflects PACU conditions. Accordingly, we conclude that the potential differences in the effects of the agents used for anesthesia maintenance should be considered in future studies and, more importantly, addressed in validation studies. 

The types of narcotic agents used represent another difference in the study designs. Contextually, the differences of the opioids used to provide general anesthesia relate to their elimination half-lives. Nevertheless, almost all eventually have the same effects on the HR. Narcotic agents inhibit sympathetic activity while preserving or increasing parasympathetic activity [35–38]. While Boselli et al. used remifentanil in both studies, demonstrating favorable findings [13,14], Ledowski et al. reported unfavorable results in their study using fentanyl as a narcotic agent [22]. It would appear, therefore, that it is more realistic to consider the preferred narcotic agent as having minimal effect on the outcome [29]. Our protocol also included the use of remifentanil, and the patients had completely overcome the effects of the applied narcotics at the time of admission to the PACU, considering that they were given time to regain spontaneous respiration after the operation.

Indeed, other than anesthetic agents, there are several factors affecting the HR at varying rates, although the effects of sex, age, awareness, varying hemodynamic and autonomic conditions, and percentage of inhaled oxygen and the interactions between these parameters are unclear [13,39–41]. Different classes of medications may also affect the HR [42], among which acetylcholinesterase inhibitors used for the reversal of neuromuscular blockages and the anticholinergic agents used to prevent their cholinergic activity are of particular importance [43,44]. Although we excluded patients using medications affecting HR from the present study, and we waited for the spontaneous resolution of neuromuscular blockage, or used a specific antagonist, sugammadex, in the presence of a clinical indication, we believe that our findings are not confounded by these factors. Accordingly, it can still be argued that the ANI may not correctly reflect autonomous system balance in routine use, given the common use of different medications with different effects on HR in daily practice. 

Generally speaking, these problems are not exclusive to the ANI. When studies investigating other methods of perioperative analgesia assessment, such as skin conductivity or surgical stress index, are reviewed, it is apparent that the relationship between these alternative methods with postoperative pain could not be proven for similar reasons [45]. 

It is necessary for us to underline some limitations of this study. Although the number of patients included in the study was comparable to previous studies, it should still be kept in mind that there were only a limited number of patients and no control group. Moreover, all of the patients fell within a certain age interval, and the study included ASA I/II and relatively healthy patients who were not receiving any concomitant medications. Indeed, the target patient population that stands to benefit from these findings is somewhat different from the patient groups in our study and previous studies. Furthermore, the study exclusion criteria, which are common in all studies, are actually a part of the daily anesthesiology routine.

In conclusion, based on the data collected in this study, we identified a weak correlation between the NRS and ANI values in patients who were not under the influence of anesthetic agents. Although, based on the information we gathered before starting this study as well as our satisfactory personal experience, we believed that ANI monitoring for anesthetized patients could at least provide alternative data on the present status of postoperative pain, the data collected in this study did not lead us to any positive conclusions. In addition, we believe that while ANI values are valuable for anesthetized patients, they cannot be used for the prediction of postoperative pain severity, contrary to the findings of other researchers. More reliable results on this matter could be obtained through controlled, randomized, and prospective studies investigating different anesthesia protocols for operations with longer durations and involving larger patient groups. We also recommend that new studies be carried out to develop an ideal analgesia-monitoring system adjusted for different scenarios in distinct patient groups, such as those at risk of insufficient pain treatment, nonverbal patients, and pediatric, geriatric, or noncooperative patients who cannot use VAS, NRS, or other conventional pain scales.
